# The effectiveness of different singly administered high doses of buprenorphine in reducing suicidal ideation in acutely depressed people with co-morbid opiate dependence: a randomized, double-blind, clinical trial

**DOI:** 10.1186/s13063-018-2843-9

**Published:** 2018-08-29

**Authors:** Jamshid Ahmadi, Mina Sefidfard Jahromi, Zahra Ehsaei

**Affiliations:** Substance Abuse Research Center, Shiraz University of Medical Sciences, Shiraz, Iran

**Keywords:** Buprenorphine, Suicidal ideation, Opioid dependence

## Abstract

**Background:**

Buprenorphine is usually administered to treat opioid use disorder and pain syndromes. This research presents the first study regarding the effectiveness of different singly administered high doses of buprenorphine (a partial opioid agonist (of μ-opioid receptors), a potent opioid antagonist (of κ-receptors) and a partial agonist of nociception receptors) in reducing suicidal ideation in acutely depressed people with co-morbid opiate dependence. It follows small studies that suggest that ultra-low-dose buprenorphine may be useful in reducing suicidal ideation. The goal of this study was to describe the outcome of different doses of buprenorphine on suicidal opioid-dependent patients over a 3-day interval, by conducting a randomized clinical trial.

**Methods:**

Fifty-one suicidal male inpatients who fulfilled the Diagnostic and Statistical Manual of Mental Disorders, Fifth Edition (DSM-5) criteria for both opioid dependence and major depressive disorder were randomized to three groups (*n* = 17 per group) to receive a single, sublingual dose of buprenorphine (32 mg, 64 mg, or 96 mg). Out of 51 participants, there were 47 patients; 16 (34.04%) received 32 mg, 17 (36.17%) received 64 mg, and 14 (29.78%) received 96 mg of sublingual buprenorphine. They were evaluated by using psychometric assessment of the Beck Scale for Suicidal Ideation (BSSI) and interviews based on DSM-5 criteria. A placebo group was not included because of the high probability of severe withdrawal without active pharmacological treatment. The study was conducted with appropriate precautions and monitoring of respiratory and cardiovascular measures. The medication was administered while the patients were in moderate opiate withdrawal, as indicated by the presence of four to five withdrawal symptoms. A structured clinical interview was conducted, and urine toxicology testing was performed.

**Results:**

Patients completed the 3-day trial course. The outcomes illustrated a significant reduction in BSSI scores within each of the three groups, *p* < 0.01., but no difference in results between the groups, *p* = 0.408.

**Conclusions:**

The results suggest that a single high dose of buprenorphine could rapidly treat suicidal ideations. A single high dose of buprenorphine may be a main-mechanism medication that gives a rapid treatment for suicidal opioid-dependent patients. Placebo-controlled trials are required to measure the safety and the physiological and psychological effects of this medication.

## Background

At the present time, suicide is a significant dilemma and requires emergency intervention. For instance, in the USA, suicide is the 10th leading cause of death in addition to being a major public health issue [1]. Most of the investigations in the field of suicide have focused on risk factors, and less concern has been addressed to promoting novel treatments for suicidal people. Usually, patients with suicidal desires are admitted to hospitals in order to prevent them from self-destructive behaviors, and then they begin counseling and/or start taking appropriate medications. A couple of weeks are required for antidepressant drugs to work, and so it is not probable to treat and control a suicidal crisis immediately [2]. Ketamine [3], lithium, and clozapine [4], prefrontal repetitive transcranial magnetic stimulation -rTMS [5], and electroconvulsive therapy -ECT [6], have been presented as acute treatments for suicidal thoughts or attempts.

Striebel et al. in 2014 applied sublingual buprenorphine for rapid dissolution of suicidal ideation in a person with treatment-resistant depression and severe opioid use disorder [7]. Yovell et al. performed a double-blind controlled clinical trial with ultra-low-dose buprenorphine for severe suicidal ideation and illuminated that ultra-low-dose buprenorphine resulted in a decline in Beck Scale for Suicidal Ideation (BSSI) scores after 2 weeks [8].

Buprenorphine is prescribed as a partial agonist of μ-opioid receptors, a potent antagonist of κ- and δ-receptors, and a partial agonist of nociception receptors [9]. The Drug Enforcement Administration (DEA) determined buprenorphine to be a schedule III drug [10], indicating that abuse could lead to moderate or low physical dependence or high psychological dependence. Accordingly, the use of buprenorphine in suicidal patients with an experience of substance abuse is challenging. Previously we discussed a case of cannabis-induced psychotic disorder and opioid depressive disorder with severe suicidal thoughts treated successfully with a single high dose (96 mg) of buprenorphine [11].

Although a number of studies indicated its reduction of suicide ideation, buprenorphine is not FDA endorsed or intended to treat suicidality. Buprenorphine is considered as potentially addictive itself. Hence it should not usually be prescribed in this situation. More studies and clinical trials are necessary to clarify this issue. Now, we are optimistic that researchers will start to build a foundation for treatment of suicidality in opioid-dependent patients [12–15].

The paper presents the first study regarding the effectiveness of different singly administered high doses of buprenorphine (a partial opioid agonist of μ-receptors, a potent opioid antagonist of κ-receptors, and a partial agonist of nociception receptors) in reducing suicidal ideation in acutely depressed people with co-morbid opiate dependence. It follows small studies that suggest that ultra-low-dose buprenorphine may be useful in reducing suicidal ideation. The postulated action of buprenorphine in reducing suicidal ideation is via the reduction of mental pain.

Currently, we are giving only a single high dose of sublingual buprenorphine as an original inlet for the rapid treatment of suicidal ideation and depression, because we theorize and contemplate that there is dysregulation of endogenous opioid function in both depression and opioid dependence [12–15]. Moreover, because buprenorphine is an agonist of μ-opioid receptors, it reduces levels of suicidal thoughts, depression, dysphoria, anxiety, pain, and opioid withdrawal symptoms. Also, because it is a powerful κ-receptor antagonist, it lessens the amount of suicidal tendencies, anxiety, and hostility [15–19].To our knowledge, there are no published controlled trials on this important affair (administration of a single high dose of buprenorphine for the treatment of suicide).

The principal goal of this trial was to examine the single dose effect of 32 mg, 64 mg, or 96 mg buprenorphine in the treatment of suicide in opioid-dependent patients.

## Methods

### Subjects

At screening, subjects were examined and questioned by a board-certified psychiatrist to determine their eligibility, i.e., “severe Opioid Use Disorder”, based on DSM-5 criteria [15]. Prior to each interview, we described the aims of the study and guaranteed confidentiality. All the patients gave written informed consent before entering the research study. The trial was approved and monitored by the Ethics Research Committee of Shiraz University of Medical Sciences, which adheres to the Declaration of Helsinki Ethical Principles for Medical Research involving human subjects.

The interviews and examinations were achieved on the premises of the treatment hospital because it appeared to be a non-threatening and suitable environment.

Patients admitted consecutively to the psychiatric inpatient ward in Shiraz city enrolled in the clinical trial. Only men were selected for the trial because only male patients are admitted to this main referral psychiatric ward. Patients were screened and interviewed to be eligible for the study. They had to meet the DSM-5 criteria for both opioid dependence and major depressive disorder [15].

Fifty-one suicidal men who fulfilled the DSM-5 criteria for both opioid dependence and major depressive disorder were randomly assigned into three groups (confidentiality was completely discussed and written informed consent was received from the men). Out of 51 patients (each group included 17 patients), four patients refused to take the single dose of buprenorphine (one from the 32-mg dose group and three from the 96-mg dose group). All of the remaining 47 patients obtained only a single high dose of buprenorphine and ended the 3-day trial time.

Suicidal ideation was questioned and assessed by comprehensive and precise interviews with the patients and the persons who accompanied them. Suicidal ideation was also measured using the Iranian translated and validated version of the BSSI [20]. Note that the patients had suicidal ideation before entering the withdrawal phase, so suicidal ideation was not due to opioid withdrawal symptoms.

Everyday opioid use for at least 1 year was a requirement. Patients were excluded if they had a substance use disorder other than opioid use. Likewise, patients who were not interested in recruitment at the start of the clinical trial were excluded.

Sublingual buprenorphine (one dose only) was administered while the patient was in moderate opioid withdrawal. The presence of two or three opioid withdrawal symptoms was considered as constituting mild withdrawal, the presence of four or five symptoms was an indication of moderate withdrawal, and the presence of six or more symptoms was an indication of severe withdrawal [15]. The buprenorphine doses tested were 32 mg, which is the maximum dosage currently used clinically, and two other doses that were twice and three times 32 mg. A placebo group was not included because of the high probability of severe withdrawal without active pharmacological treatment.

### Randomization

In a double-blind manner the patients were randomly placed in one of the three treatment groups. We used a standard randomization procedure produced by computer to obtain a random sample set.

### Procedure

The research team was adequately trained and included an addiction psychiatrist, a general psychiatrist, a general practitioner, a psychologist, a nurse, and a statistician.

The pills all had the same shape and color. The patients and the research staff were blind to the medications for the period of the study. The ratings and interviews were done by a fully trained physician who was unaware of the medications and adverse events.

A psychometric assessment using the BSSI was administered to the inpatients to monitor the level of suicidal ideation [20] before the development of opioid withdrawal symptoms. Patients randomly obtained 32 mg, 64 mg, or 96 mg of buprenorphine as a single high dose only and were admitted to a psychiatric ward. Since the patients would develop severe withdrawal symptoms if they were not administered buprenorphine, we did not include a placebo control group. Out of 47 patients, 16 (34.04%) received 32 mg, 17 (36.17%) received 64 mg, and 14 (29.78%) received 96 mg of buprenorphine. The patients received buprenorphine (only a single dose) when they developed moderate opioid withdrawal symptoms. The patients were followed for 3 days. The outcome was monitored and measured by daily scoring of suicidal ideation according to the BSSI and also DSM-5 criteria for major depression.

Although our inpatient facility was a controlled environment, for more accuracy and precision, a urine drug screening test via thin-layer chromatography (TLC) was performed. To ensure safety, adverse effects, vital signs, respiration, and gastrointestinal effects were monitored every hour for the first day and then every 6 h.

We advocate using a single dose on an inpatient basis and then having the patients released drug-free without medication assistance and with an appointment for close psychosocial follow-up.

### Statistical analysis

Data analysis was administered using SPSS version 18. Analysis of variance (ANOVA) and Student *t* test analyses were used to examine for differences in means, and chi-square analyses were used to test for differences in frequencies. Two-sided tests were used at 0.05 levels.

## Results

**Fig. 1:**
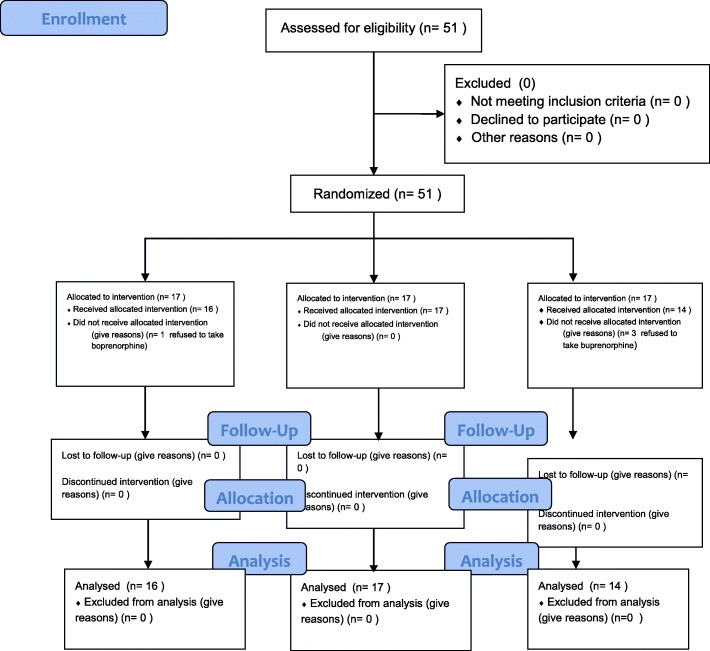
Consolidated Standards of Reporting Trials (CONSORT) flowchart of the patients in this trial

**Fig. 2:**
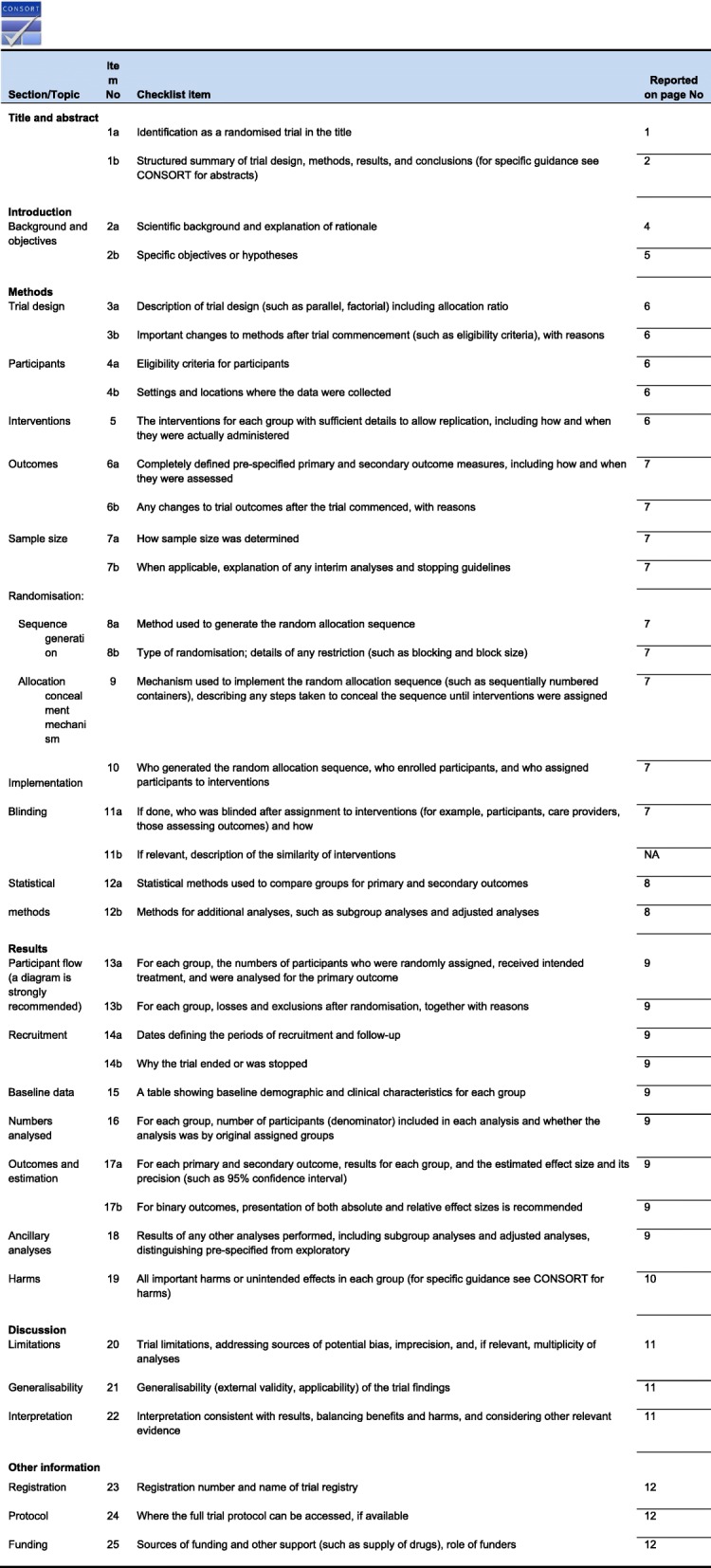
CONSORT 2010 checklist of information to include when reporting a randomized trial

The Consolidated Standards of Reporting Trials (CONSORT) process flowchart and a checklist are shown in Figs. 1 and 2. Fifty-one patients were screened to enter this trial. Seventeen patients were randomly allocated into one of the three groups. Out of 51 patients, four patients refused to take the single dose of buprenorphine (one from the 32-mg buprenorphine group and three from the 96-mg buprenorphine group). All 47 patients obtained only a single high dose of buprenorphine and completed the 3-day trial time. Thus, the data were gathered from 47 opioid-dependent men whose mean age was 32.87 ± 7.50.

Out of the 47 patients, 16 (34.04%) received 32 mg, 17 (36.17%) received 64 mg, and 14 (29.78%) received 96 mg of buprenorphine. Patients received buprenorphine (a single dose) when they developed moderate opioid withdrawal symptoms.

During the course of the study, no illicit opioid use was detected (based on everyday interviews and urine toxicology tests.

The three groups did not differ regarding demographic characteristics (Table 1). Table 2 displays the suicide ideation scores of the three groups during the 3 days of treatment interval. As we observe in the 32-mg dose group, there are significant statistical differences in suicide ideation scores from day 1 to day 3 (*p* < 0.01). There are also significant differences in scores from day 1 to day 3 in both the 64-mg dose group (*p* < 0.01) and the 96-mg dose group (*p* < 0.01).

**Table 1:** Demographic characteristics of the patients

Group		32 mg *N* = 16 (34.04%)	64 mg *N* = 17 (36.17%)	96 mg *N* = 14 (29.78%)	Total *N* = 47	Chi-square	df	*p* value	*F*
Age (years)		31.75 ± 6.40	33.94 ± 9.01	32.85 ± 7.00	32.87 ± 7.50		2	0.713	0.341
Duration of drug abuse (years)		8.81 ± 5.45	12.00 ± 7.53	10.57 ± 6.28	10.48 ± 6.51		2	0.380	0.988
Job	Unemployed	6 (37.5)	12 (70.6)	3 (21.4)	21 (44.7)	12.708	6	0.052	
	Self-employed	10 (62.5)	3 (17.6)	10 (71.4)	23 (48.9)				
	Employed	0 (0)	1 (5.9)	1 (7.1)	2 (4.3)				
Education	Illiterate	1 (6.3)	0 (0)	0 (0)	1 (2.1)	7.668	8	0.467	
	Primary school	5 (31.3)	4 (23.5)	5 (35.7)	14 (29.8)				
	High school	6 (37.5)	11 (64.7)	7 (50)	24 (51.1)				
	Higher education	4 (25)	2 (11.8)	1 (7.1)	7 (14.9)				
Marital status	Single	10 (62.5)	6 (35.3)	10 (71.4)	26 (55.3)	5.600	4	0.231	
	Married	6 (37.5)	10 (58.8)	4 (28.6)	20 (42.6)				
	Divorced	0 (0)	1 (5.9)	0 (0)	1 (2.1)				

*df* Degrees of Freedom, *F* F value in F-test for analysis of variance (ANOVA)

**Table 2:** BSSI means

Group	Day	32 *N* = 16 (34.04%)	64 *N* = 17 (36.17%)	96 *N* = 14 (29.78%)	*F*	Df	*p* value
Baseline		8.50 ± 8.53	11.05 ± 9.58	8.24 ± 6.08	0.528	2	0.594
Day 1		3.81 ± 7.79	4.82 ± 8.05	1.64 ± 3.05	0.845	2	0.436
Day 2		1.25 ± 2.88	3.35± 7. 14	0.714 ± 2.67	1.322	2	0.277
Day 3		0.625 ± 2.50	1.17 ± 4.85	0.00 ± 0.00	0.497	2	0.612
*F*		150.507	11.960	27.827			
*p* value		0.00	0.00	0.00			
Df		3	3	3			
Power		1.00	0.999	1.00			
Total of days		3.54 ± 5.20	5.10 ± 6.21	2.69 ± 2.77	0.915	2	0.408

*F* F value in F-test for analysis of variance (ANOVA), *Df* Degrees of Freedom

**Fig. 3:**
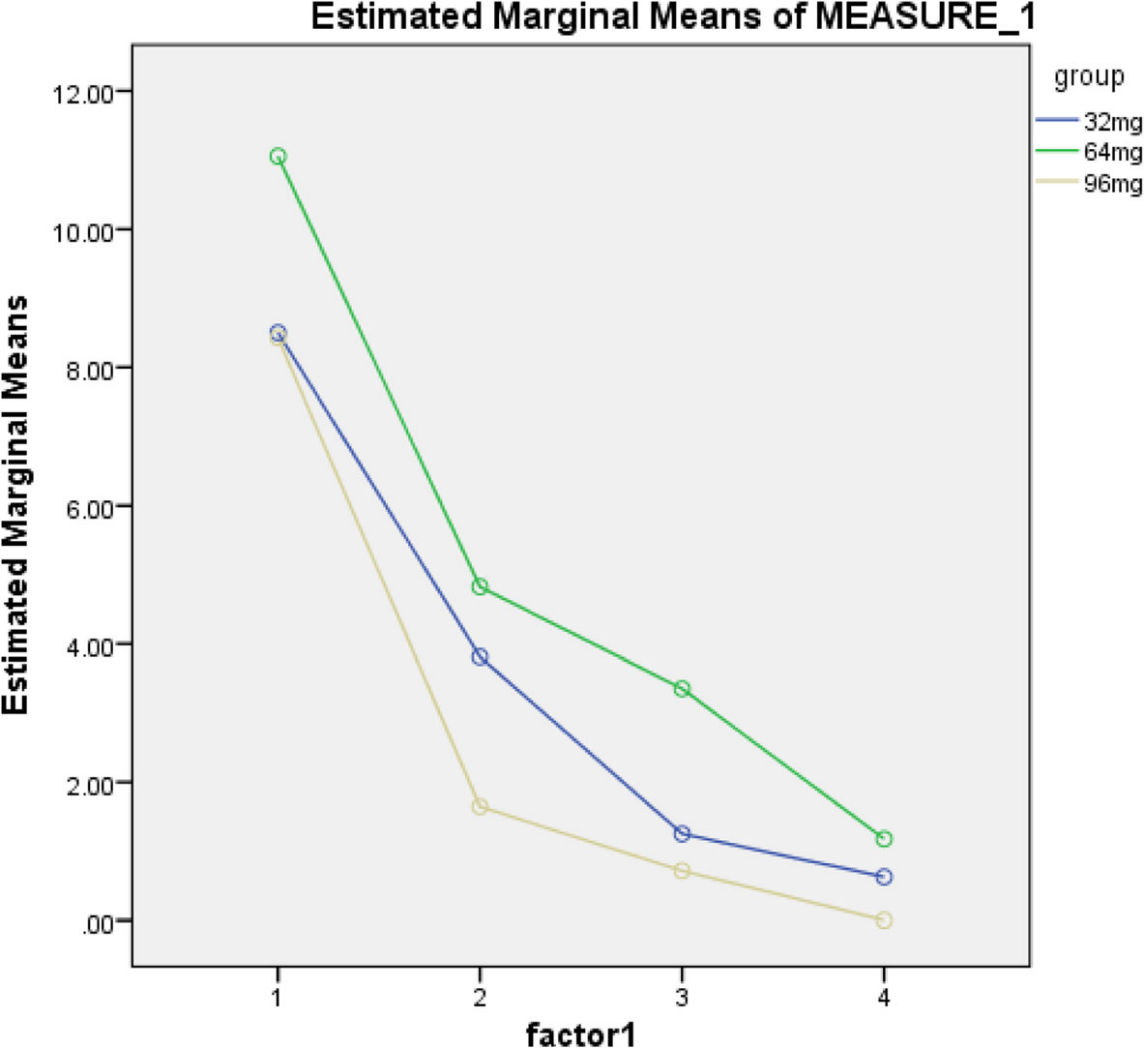
Beck Scale for Suicidal Ideation (BSSI) results for the three groups during the 3 days following buprenorphine administration (repeated measures)

Comparing the mean suicide scores across the groups, we cannot find any significant differences, *p* = 0.408. Figure 3 represents the BSSI results from day 1 to day 3 in all three groups. We followed up with the patients 2 weeks later while they attended an outpatient clinic, and none of them experienced suicidal ideation.

### Adverse events

Based on our protocol, all 47 participants were managed in the same way. Sublingual buprenorphine (as a single dose only) was administered while the opioid use disorder patients were moderately in withdrawal. The study was conducted with appropriate precautions and monitoring of respiratory and cardiovascular measures. Four patients (one from the 32-mg, one from the 64-mg, and two from the 96-mg dose group) experienced significant hypotension, nausea, or vomiting. They were managed with antiemetic medications or hydration. There were no significant drug adverse effects or drug intolerance in the other patients. There was no report of rebound after this trial.

Drug adverse effects were questioned, monitored, and measured by precise interviews three times a day.

## Discussion

Buprenorphine has been considered as a plausible antisuicidal, antidepressive, and antianxiety medication in patients with opioid use disorder, mood disorder, or anxiety disorder. A likely pharmacological reason for the antisuicidal and antidepressive influence of buprenorphine is that the medication is not only an agonist of the μ-opioid receptor but also a powerful antagonist of the κ-opioid receptor [21–39].

The κ-receptor and its ligand dynorphin seem to function in the advancement of dependence disorders [40] in addition to the progression of depressive and anxiety disorders. κ-antagonists have antisuicidal, antidepressive, and antianxiety effects. Research studies have revealed that activation of dynorphin is likely accompanied by negative emotional conditions, anxiety, and depression [40]. Furthermore, in the rat model, administering of κ-opioid receptor agonists can provoke depressive situations [41].

This work showed that a single high dose of buprenorphine seems to be clinically effective and safe. Our research advises that this single high dose may also provide a rapid, simple, and safe means of treating suicide ideation. Administration of a single high dose of buprenorphine appears to diminish concerns about compliance, dependence, diversion, and abuse. Furthermore, the cost considerations appear to be suitable, particularly when we explore the possibility of administration for outpatient individuals without a requirement for hospital admission. However, we mention that, still, this is a feasibility study.

We also note that having a patient with a substance use disorder withdraw from the hospital under supervision and then making him an appointment for psychosocial follow-up, thus allowing him to return to his supportive family, often happens in Iran.

The strengths of this study included the randomized clinical trial design, the careful diagnosis process using DSM-5 criteria and urine toxicology, and the result of patients who did not experience suicidal ideation 2 weeks later while they attended an outpatient clinic. However, the study had some limitations. The results of the current study need to be replicated with (1) a larger sample including women, (2) a longer duration, and (3) a sample including a placebo group.

## Conclusions

Our findings illustrated a significant decline in suicide scores within each of the three groups but no difference in results between the groups. Furthermore, none of the participants experienced suicidal ideation at the end of the 3-day trial and 2-week follow-up. A single high dose of buprenorphine could be a novel-mechanism drug that offers rapid treatment for suicidal thoughts and major depressive disorders in opioid-dependent patients. Placebo-controlled trials of longer duration are necessary to present the power, safety, psychological, and physiological effects of extended exposure to this medication.

## Abbreviations

BSSI: Beck Scale for Suicidal Ideation
DSM-5: Diagnostic and Statistical Manual of Mental Disorders, Fifth Edition
ECT: Electroconvulsive therapy
RCT: Randomized clinical trial
rTMS: Prefrontal repetitive transcranial magnetic stimulation
